# DNA Methylation Demonstrates Bronchoalveolar Cell Senescence in People Living with HIV: An Observational Cohort Study

**DOI:** 10.3390/biomedicines12061261

**Published:** 2024-06-06

**Authors:** Ana I. Hernandez Cordero, Xuan Li, Julia Yang, Chen Xi Yang, Tawimas Shaipanich, Julie L. MacIsaac, Kristy Dever, Michael S. Kobor, Julio Montaner, Marianne Harris, Silvia Guillemi, Shu Fan Paul Man, Don D. Sin, Janice M. Leung

**Affiliations:** 1 Centre for Heart Lung Innovation, St. Paul’s Hospital, University of British Columbia, Vancouver, BC V6Z 1Y6, Canada; 2 Edwin S. H. Leong Centre for Healthy Aging, University of British Columbia, Vancouver, BC V6T 1Z3, Canada; 3 Division of Respiratory Medicine, Department of Medicine, Faculty of Medicine, University of British Columbia, Vancouver, BC V6Z 1Y6, Canada; 4 Centre for Molecular Medicine and Therapeutics, University of British Columbia, Vancouver, BC V6H 0B3, Canada; 5 British Columbia Centre for Excellence in HIV/AIDS, Vancouver, BC V6Z 1Y6, Canada; 6 Department of Medicine, Faculty of Medicine, University of British Columbia, Vancouver, BC V6Z 1Y6, Canada; 7 Department of Family Practice, Faculty of Medicine, University of British Columbia, Vancouver, BC V6Z 1Y6, Canada

**Keywords:** BAL, HIV, DNA methylation, aging, PLWH

## Abstract

Background: DNA methylation may be a link between HIV, aging, and the increased risk of lung comorbidities. We investigated whether bronchoalveolar lavage (BAL) cells of people living with HIV (PLWH) demonstrate epigenetic disruptions and advanced epigenetic aging. Methods: BAL cell DNA methylation from 25 PLWH and 16 HIV-uninfected individuals were tested for differential methylation of Alu and LINE-1 sites, markers of aging. We used a weighted gene correlation network analysis to identify HIV- and age-associated co-methylation networks. We tested the effect of HIV on DNA methylation using a robust linear model (false discovery rate < 0.10). Results: The BAL cells of PLWH were marked by global hypomethylation in both Alu and LINE-1 elements. Six co-methylated CpG networks were identified that were significantly associated with age; of these, the red module was significantly differentially methylated in PLWH and enriched pathways (e.g., Ras signaling and T-cell receptors). We identified 6428 CpG sites associated with HIV. Conclusions: We have shown here for the first time that alterations in the DNA methylation of BAL cells in the lung with HIV show a pattern of advanced aging. This study strongly supports that HIV may contribute to an increased the risk of lung comorbidities through the epigenetics of aging.

## 1. Introduction

People living with human immunodeficiency virus (HIV, PLWH) are at high risk of developing chronic diseases, despite the therapeutic benefits of antiretroviral therapy (ART) [1,2,3]. The use of ART has led to a decrease in the incidence of opportunistic lung infections in PLWH, nonetheless chronic lung conditions remain a considerable burden in this population [4]. Although PLWH are living longer [5], age-related comorbidities develop earlier compared to the general public, suggesting that the virus or its treatment accelerates biological aging [6]. The lung is one of the organs most impacted by this phenomenon. HIV negatively affects aging mechanisms in the lung such as oxidative stress and cellular senescence [7], which may ultimately place PLWH at higher risk for chronic obstructive lung disease (COPD) or lung cancer. The early targeting of these mechanisms may potentially reduce the risk of comorbidities and poor clinical outcomes in this population.

One of the best indications of a cell’s biological age is its DNA methylation profile. DNA methylation is an epigenetic mechanism that involves the addition of a methyl group to a cytosine base located next to guanine base (CpG site). As a dynamic alteration, it can be affected by acquired infections such as HIV [8]. DNA methylation is tissue-specific and at promoter regions is associated with changes in gene expression [9], which can potentially affect downstream phenotypes. We have shown, for instance, that the blood and airway epithelial epigenomes are altered in PLWH compared to HIV-negative individuals [8,10], and that these epigenetic alterations are consistent with a pattern of accelerated aging [10,11]. Previous findings by our laboratory have also suggested that the blood of PLWH with airflow obstruction is characterized by global hypomethylation [12]. Global DNA methylation can be measured through the methylation of ubiquitous transposable elements Alu and LINE-1 that are abundant throughout the genome; hypomethylation along these elements is associated with advanced aging [13] and disease [14]. While we have reported these findings in airway epithelial and blood cells, whether HIV alters DNA methylation in bronchoalveolar lavage (BAL) fluid cells (predominantly alveolar macrophages) to promote accelerated aging has not yet been reported. Here, we hypothesized that HIV induces epigenetic disruptions in the BAL cells present in the airway along accelerated aging pathways. 

## 2. Materials and Methods

### 2.1. Study Cohort

Study participants included 41 adults, of which 25 were people living with HIV (PLWH), and 16 were HIV-uninfected participants. This cohort has been previously described [10,11]. Briefly, all participants underwent flexible bronchoscopy according to standard procedures previously published [15]. BAL samples were obtained by instilling aliquots of sterile saline into the right middle lobe or lingula until a return volume of 30 mL or total instilled volume of 200 mL was reached [16]. All participants underwent pre-bronchodilator spirometry according to American Thoracic Society guidelines [17]. The samples were obtained between 2015 and 2019 at St. Paul’s Hospital in Vancouver, British Columbia, Canada. This study was approved by the University of British Columbia Research Ethics Board (Certificates H11-02713 and H15-02166). All participants provided written informed consent.

### 2.2. DNA Methylation Profiling

DNA was extracted from BAL cells using the DNeasy Blood and Tissue Kit (Qiagen, Hilden, Germany) and the unmethylated cytosine residues were converted to uracil using the EZ DNA methylation kit (Zymo, Irvine, CA, USA). The extracts were profiled for DNA methylation using the Illumina Infinium MethylationEPICv1 BeadChip microarray which captures 863,904 CpG sites. Samples were randomized by age, sex, and HIV status on the chips to minimize batch effects. The β-values for each CpG probe were calculated as the ratio of methylation intensity to the overall intensity. β-values range from 0 (fully unmethylated) to 1 (fully methylated) and were log transformed to M-values for the downstream analyses. Preprocessing and quality control steps for the DNA methylation data included background correction and normalization using the normal-exponential out-of-band [18] and β-mixture quantile normalization [19], respectively. No batch effects were detected. This pipeline has been previously standardized by our laboratory [8,12,20,21].

The profiles were used to infer ancestry using the EPISTRUCTURE software (version GLINT_1.0.4) [22] and the first 5 principal components were retained for downstream analyses. We later selected variables to be included in our downstream analyses as follows. The BAL cell DNA methylation profiles were used to conduct a principal component analysis (PCA). We then calculated the correlation between the first two DNA methylation principal components (PC1 and PC2) and demographic characteristics (smoking, age, sex, body mass index [BMI], BAL macrophage percentage, pre-bronchodilator forced expiratory volume in 1 s (FEV_1_)% predicted, pre-bronchodilator FEV1/forced vital capacity (FVC) ratio, COPD status, and 5 PCs [EPISTRUCTURE]). Variables with significant (*p* < 0.05) correlations were included as covariates in the differential methylation analyses (age, sex, smoking status, and PC1 and PC2) (Figure S1). BAL macrophage percentage was also included in the differential methylation analyses because cell type proportions may affect DNA methylation [23].

### 2.3. Alu and LINE-1 Imputation

Global hypomethylation is an accepted measure of biological age, measured by methylation at Alu and LINE-1 repetitive elements [24,25]. The repetitive element methylation prediction machine learning tool (R package REMP, version 4.4) [26] was used to impute DNA methylation levels at Alu and LINE-1 sites based on the BAL DNA methylation profiles. We later conducted a robust linear model (rlm) using the MASS R package version 7.3-60.2 (M-estimation) to test the association between HIV status and global methylation. The resulting model was the following: M-value~HIV status + age + sex + smoking status + BAL macrophage percentage + PC1 + PC2. To determine whether COPD status was also associated with global hypomethylation, we repeated this test using the following model: M-value~COPD status + age + sex + smoking status + BAL macrophage percentage + PC1 + PC2. Significant effects were determined at a false discovery rate (FDR) < 0.10. 

### 2.4. Weighted Gene Correlation Network Analysis (WGCNA)

Analytical approaches that treat each CpG as an independent unit of analysis may overlook coordinated CpGs that share a common regulatory mechanism or participate in similar biological processes and pathways. To identify co-methylated networks associated with aging and HIV, we used a weighted gene correlation network analysis (WGCNA) approach [27]. This method has been previously used to identify co-methylated CpG sites [28]. Briefly, we selected genome-wide CpGs annotated to promoter regions based on the Methylation Consortium (https://clockfoundation.org/mammalianmethylationconsortium/ accessed on 20 August 2023); in total, we retained 105,712 CpG sites. We used the WGCNA R package (version 1.72-5) to construct networks of co-methylated CpGs (modules) using M-values. Key measures obtained from this analysis included the module’s eigengene (summarized methylation for a group of co-methylated sites, i.e., the principal component of the module’s methylation); and the CpGs module membership (the correlation between methylation M-value and eigengene). We used Pearson’s correlations and univariate linear models to test the association between each of the modules’ eigengenes and age, HIV status, sex, macrophages percentage, CD4T count, smoking status, COPD, and FEV_1_% predicted (FDR < 0.10) in a hypothesis-free approach. First, we identified modules whose eigengenes were significantly correlated with age at FDR < 0.10. Next, we identified whether these modules were differentially methylated in PLWH compared to HIV-uninfected participants after adjustment for age, with significance set at *p* < 0.05.

### 2.5. Differential Methylation Analysis

We conducted an epigenome-wide analysis to identify differentially methylated CpG positions or DMPs associated with HIV status using the MASS R package [29]. The rlm used was adjusted for age, sex, smoking status, macrophage percentage, and the first two EPISTRUCTURE PCs. Significant DMPs were defined at an FDR < 0.10.

### 2.6. Pathway Enrichment Analysis

We identified Kyoto Encyclopedia of Genes and Genomes (KEGG) pathways that were enriched by genes that corresponded to CpG sites identified in the WGCNA and differential methylation analysis. The R package WebGestalt 2019 [30,31] was used for this analysis. Significant enrichment was defined at FDR < 0.10.

## 3. Results

### 3.1. Study Overview

Table 1 shows the demographic and clinical characteristics of our study cohort. PLWH were significantly younger, had a larger proportion of males, and lower lung function (based on pre-bronchodilator FEV_1_/FVC) than HIV-uninfected participants. BMI, smoking status, BAL macrophage percentage, and inhaled corticosteroid treatment were similar between the two groups, however, there was a higher proportion of physician-diagnosed COPD within PLWH. The majority of PLWH were on ART and had undetectable HIV viral loads. BAL cells were predominantly macrophages as expected.

### 3.2. Global Hypomethylation Is Observed in the BAL Cells of PLWH, but Not in COPD

**Figure 1:**
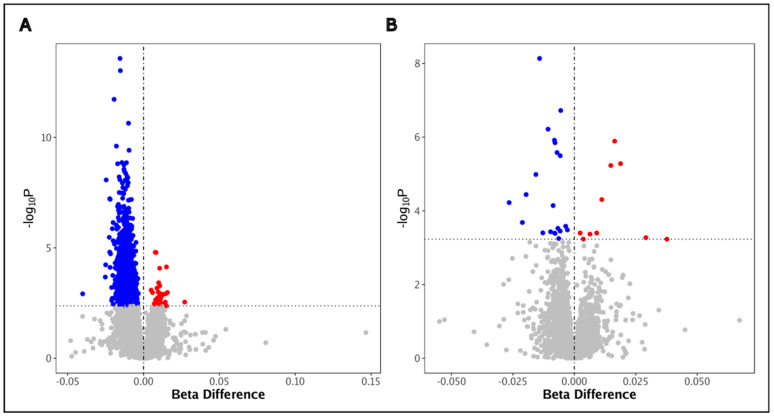
Global hypomethylation was observed in the bronchoalveolar lavage cells of PLWH. Markers of global methylation (**A**) Alu and (**B**) LINE-1 identified in BAL cells at FDR < 0.10 (level of significance is represented by the horizontal grey dotted line). The *x*- and *y*-axes represent the beta difference effect size and level of significance, respectively, for the association between each Alu and LINE-1 site and HIV. Blue = hypomethylation in PLWH compared to HIV-uninfected individuals; Red = hypermethylation in PLWH compared to HIV-uninfected individuals. The horizontal dotted line represents the line of statistical significance.

Our analyses on the retro-transposable elements Alu and LINE-1 demonstrated a signature of global hypomethylation in BAL cells that was associated with HIV. Figure 1A shows that the majority of the significant Alu sites were found to be hypomethylated in PLWH (2259 out of 2294 sites). A similar pattern of hypomethylation (although less evident) was identified in LINE-1 sites (21 out 31 sites) (Figure 1B). In contrast, we did not find an association between global hypomethylation and COPD. In total, 14 Alu sites were associated with COPD, of which only 7 demonstrated hypomethylation (Figure S2), thus no pattern of hypomethylation was identified. A total of 11 LINE-1 sites were associated with COPD, of which 10 showed hypermethylation (Figure S3).

### 3.3. Age-Associated Co-Methylation Networks Are Dysregulated in HIV

We hypothesized that epigenetic networks involved in the progression of aging are altered in PLWH. We conducted a WGCNA analysis on CpGs within promoter regions of the genome, yielding 77 co-methylated modules (Table S1). Through a univariate linear model, we determined that age was associated with the eigengene of six modules at FDR < 0.10. Table 2 shows the modules that had significant correlations with age; no other co-methylation modules were associated with age. The yellow module demonstrated the strongest correlation with age (R = −0.61), while the pink had the weakest correlation out of the six modules (R = −0.45).

**Figure 2:**
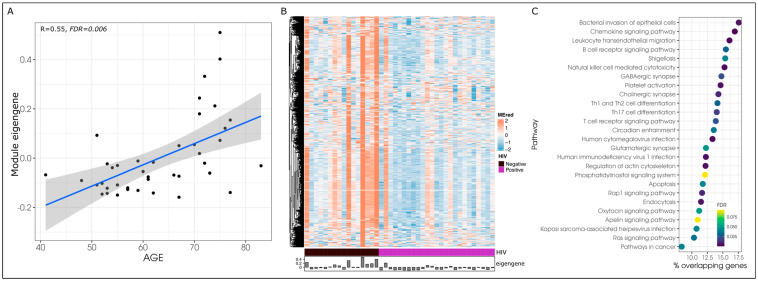
Co-methylation network identified in BAL cells was associated with age and HIV. (**A**) Linear relationship between age and the red module co-methylation network eigengene is represented by the blue line with the grey area representing the 95% confidence interval for the linear relationship); black dots represent the module eigengene and chronological age values for each participant. (**B**) Heatmap of M-values of CpGs within the red module co-methylation network (*y*-axis) by HIV status (purple = PLWH, black = HIV-negative). The eigengene value is represented in the bottom bar. (**C**) Differentially methylated pathways (Kyoto Encyclopedia of Genes and Genomes) enriched by co-methylated CpGs (co-methylation network) associated with age and HIV (FDR < 0.10).

We then hypothesized that epigenetic networks involved in the progression of aging are altered in PLWH. To investigate this hypothesis, we focused on the six age-associated modules and tested whether HIV has a significant effect on each module eigengene. We found that HIV was associated with all six age-associated modules (*p* < 0.05), of which the red module remained significant after adjusting for the effect of age (*p* = 0.044) (Table 2). The linear relationship between age and the red module (Figure 2A) had a correlation of R = 0.55 (*p* = 0.006), however, this relationship appears to be more evident in the HIV-uninfected group (R = 0.45, *p* = 0.079) compared to PLWH (R = 0.27, *p* = 0.193). Overall, PLWH were characterized by the hypomethylation of CpGs within the red module compared to HIV-uninfected participants (Figure 2B).

This table shows the number of CpGs (co-methylated sites) and corresponding genes within each age- and HIV-associated co-methylation network (module). The level of significance (*p*-value and false discovery rate [FDR]) corresponds to the association between each module’s eigengene (or first principal component) and age and HIV. HIV tests were further adjusted for chronological age. 

Further exploration of the red module showed that its CpGs demonstrated a module membership (correlation between M-value and eigengene [i.e., highly interconnected CpGs]) that ranged from 0.29 to 0.99. The top 10 CpGs based on their module membership within the red module corresponded to the following protein coding genes: *SH2D7*, *PKN1*, *PIK3CG*, *CD58*, *MYO9B*, *PTPN22*, *ARHGAP15*, *TESPA1*, *DCK*, and *MOB1B*; (Table S2). The top 10 CpGs located within a CpG island were *ITGB4*, *FOXP4*, *RP11-750H9.5*, *S1PR4*, *ITGA4*, *ICAM1*, and *RASSF1*. The red module significantly enriched 26 pathways (FDR < 0.10), including the human immunodeficiency virus 1 infection, Ras signaling, T-cell receptor, and bacterial infection of epithelial cells pathways, amongst others (Figure 2C). No other demographic, disease, or laboratory phenotype demonstrated a significant association with the red module.

### 3.4. HIV Is Associated with DNA Methylation Alterations in BAL Cells

**Figure 3:**
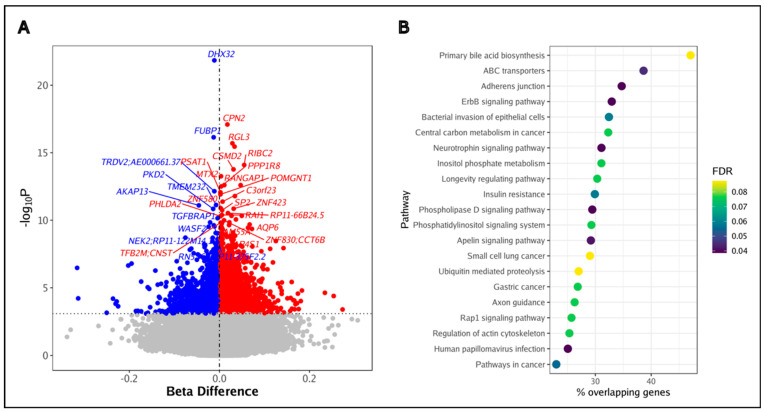
Differential DNA methylation in BAL cells associated with HIV. (**A**) Epigenome-wide differential methylation associated with HIV (FDR < 0.10). The *x*- and *y*-axes represent the effect size and level of significance (represented by the horizontal grey dotted line), respectively, for the association between each methylation site and HIV. Blue = hypomethylation in PLWH compared to HIV-uninfected individuals; Red = hypermethylation in PLWH compared to HIV-uninfected individuals, horizontal dotted line represents the level of significance based on FDR < 0.10. (**B**) Differentially methylated pathways (Kyoto Encyclopedia of Genes and Genomes) associated with HIV (FDR < 0.10).

Our epigenome-wide analysis yielded 6428 DMPs (Table S3, Figure 3A) in BAL cells that were associated with HIV (FDR < 0.10). Their methylation beta difference ranged from −0.316 to 0.273 (a beta difference of 1 corresponds to 100% methylation in PLWH vs. 0% methylation in the HIV-uninfected group while a beta difference of −1 corresponds to 0% methylation in PLWH vs. 100% methylation in the HIV-uninfected group). Table 3 shows the top DMPs based on two criteria: smallest FDR and largest beta difference effect size. The 6428 DMPs associated with HIV corresponded to 4713 DMGs. The top differentially methylated genes that corresponded to the DMPs with highest statistical significance included *DHX32*, *CPN2*, *FUBP1*, *RGL3*, and *OTOG.* The DMPs with the largest effect sizes corresponded to *ABCB11*, *MYOM2*, *PARP12*, and *7SK*. 

The DMGs significantly enriched (FDR < 0.10) 21 KEGG pathways including bacterial invasion of epithelial cells, central carbon metabolism in cancer, longevity regulating, Insulin resistance, apelin signaling, small cell lung cancer, Rap1 signaling, and pathways in cancer, amongst others (Table S4, Figure 3B). We compared our findings to previous methylation data in PLWH [8,10] and found that approximately 32% and 60% of DMGs were also identified in the blood immune cells and in the airway epithelial cells, respectively (Table S5). While only 10% of HIV-associated pathways identified in BAL cells overlapped with those identified in blood, 76% of HIV-associated pathways identified in BAL cells were also found in the airway epithelial cells of PLWH (Table S6). These include aging-related pathways such as Rap1 signaling, the longevity regulating pathway, and insulin resistance. 

## 4. Discussion

This is the first report to demonstrate that BAL cells in HIV have numerous epigenetic alterations in a pattern suggestive of accelerated aging. Our study revealed four main observations: first, these cells exhibited global hypomethylation as detected in Alu and LINE-1 elements along the genome, indicative of more advanced aging in the lungs of PLWH; second, we discovered numerous epigenetic networks of co-methylated CpGs in these cells that were associated with age, of which the red module was also significantly altered in PLWH; third, the inflammatory cells (mainly alveolar macrophages) in the airways of PLWH are characterized by epigenome-wide disruptions; and fourth, these epigenetic alterations are enriched along multiple biological processes that are known to be associated with aging and cancer, potentially linking DNA methylation with the known pulmonary complications associated with HIV. Together, these results demonstrate a pattern of epigenetic age alterations in BAL cells that complements what we have previously described in the blood and airway epithelium [10].

Our findings suggest that despite immune reconstitution and suppressed HIV viral loads, BAL cell methylation may still be impaired in PLWH. Although previous studies have shown that ART can deaccelerate HIV-associated aging through epigenetic mechanisms [32], complete reversion to pre-seroconversion aging rates similar to those observed in uninfected populations may not yet be realized. These persistent changes, combined with the alterations we have recently observed in airway epithelial cells [10], raise the possibility that they may drive the onset of age-related pulmonary comorbidities in PLWH such as COPD or lung cancer. Furthermore, blood senescence in PWLH demonstrated by advance blood epigenetic age [8,12], together with our present findings of BAL cells global hypomethylation, further support a widespread immunosenescence in PLWH. We propose that linking these BAL methylation changes in a larger cohort to disease outcomes in this population would be a fitting next step, allowing for a better understanding of how global accelerated aging across multiple lung compartments might result in injury. 

Our analyses highlighted genes with potential links to HIV pathogenesis. For instance, we found multiple hypomethylated DMPs within the promoter region of *RUFY1*, which are located within a CpG island region (regions of the genome rich in CpG sites that are often associated with changes in gene expression). Our previous methylation work in blood and airway epithelial cells of PLWH also yielded significant DMPs within *RUFY1*, suggesting that this particular gene may be systemically epigenetically disrupted in HIV [8,10]. The inactivation of *RUFY1* inhibits efficient recycling of endocytosed transferrin [33,34,35], one of the mechanisms that has been implicated in the internalization of HIV-1 [36]. *RUFY1* has been associated with time on ART in a previous trial investigating a therapeutic HIV-1 vaccine and romidepsin (a latency reversing agent) on HIV viral load, where a late ART start was correlated with blood DNA hypomethylation at *RUFY1* [37]. Although we are unable in our work to connect whether BAL cell hypomethylation in this gene was related to the timing of ART initiation, we speculate that epigenetic disruptions that occur in relation to sub-optimal ART timing may also occur in the lung. 

Our research also revealed other potential epigenetically regulated mechanisms of lung pathogenesis in HIV. We note, for example, the significant enrichment in HIV-associated DMPs for the bacterial invasion of epithelial cells pathway which may indicate that the innate immune defense of the lung may be compromised in PLWH through DNA methylation. Despite the advances of ART, bacterial pulmonary infections remain a common comorbidity in PLWH [38,39]. Previous research has demonstrated that HIV can be recovered from BAL macrophages [40,41,42], and that HIV impairs the alveolar macrophage defense against pneumococcal infection through impaired apoptosis and mitochondrial reactive oxygen species processes [42]. We raise the possibility that these cellular impairments may in part be epigenetically regulated, although further work would be required to explore this hypothesis. 

Although our findings show important and novel observations for PLWH, this study is not without its limitations. First, although our bronchoscopy study cohort offers many unique advantages to investigate the lung-specific effects of HIV, our sample size was limited due to the invasive nature of the procedure and may not reflect the totality of this population. In addition, differences between PLWH and uninfected participants, such as smoking status and COPD prevalence, may have affected our conclusions; however, we adjusted our analysis for potential confounding to the best of our ability. The influence of other age-related comorbidities would also need to be tested in a larger cohort. Stratification by duration of HIV, duration of ART, viral control, and comorbidities was not able to be performed, but would yield greater insight into differential airway immune cell aging in the wider population of PLWH. Second, BAL samples contain multiple types of immune cells and the effect of HIV on specific cell types may vary. Although our analyses were controlled for cell proportions, our profiles reflect an average methylation across all cells. Future work using single cell sequencing or flow cytometry would help us understand the cell-specific aging process that occurs in HIV. Third, it remains unclear how HIV may affect the lung epigenome over time as our work was cross-sectional. Whether the BAL cells begin to age rapidly after HIV seroconversion or whether we are observing instead a gradual accumulation of epigenetic disruptions over time is still unclear. Future research should focus on the serial profiling of PLWH in order to investigate the longitudinal effects of HIV. Finally, we were not able to link our findings to specific clinical outcomes or downstream effects in PWLH. Cohorts followed longitudinally for lung function decline, incident pulmonary disease, and respiratory-related events would help us understand the clinical implications of accelerated BAL cell aging. 

## 5. Conclusions

Notwithstanding the limitations of this study, we have demonstrated that HIV may be linked with genome-wide epigenetic disruptions and aging in the lung’s BAL cell compartment, although further work will be needed to decode the contributions of smoking and COPD to these observations. Together with our previous findings in the airway epithelium of PLWH, these results give support to the idea of global lung aging in HIV. Efforts to understand the etiology of and strategies to mitigate this aging process are imperative to improving lung health in PLWH.

## Figures and Tables

**Table 1:** Study cohort characteristics.

	PLWH	HIV-Uninfected	*p*
n	25	16	-
Male Sex (%)	92	56	0.016
Age, years	57.00 (53.00–62.00)	72.00 (70.00–75.00)	7.844 × 10^−4^
BMI, kg/m^2^	24.54 (22.02–27.91)	26.07 (24.66–29.41)	0.279
Pre-bronchodilator FEV_1_, L	2.650 (2.06–3.06)	2.490 (2.08–2.65)	0.310
Pre-bronchodilator FVC, L	4.07 (3.51–4.71)	3.24 (2.87–3.74)	0.010
Pre-bronchodilator FEV_1_/FVC (%)	62.20 (54.93–74.94)	74.13 (68.44–76.62)	0.048
Pre-bronchodilator FEV_1_% predicted	77.00 (65.00–91.00)	87.00 (74.25–96.75)	0.195
Pre-bronchodilator FEV_1_/FVC % predicted	93.10 (81.00–105.00)	92.00 (74.50–107.65)	0.820
Smoking status			0.258
Current smoker, (%)	36	12	
Former smoker, (%)	56	69	
Never smoker, (%)	8	19	
Smoking pack-years	30 (11.50–45.00)	30 (12.50–37.50)	0.450
Physician-diagnosed COPD, (%)	76	25	0.003
Bronchiectasis, (%)	4	0	~1
BAL neutrophils, (%)	2.00 (1.00–5.50)	3.63 (1.19–6.38)	0.462
BAL lymphocytes, (%)	2.50 (1.50–5.50)	2.75 (1.00–3.75)	0.758
BAL eosinophils, (%)	0.25 (0.00–1.00)	0.63 (0.23–4.06)	0.041
BAL macrophage, (%)	92.50 (79.50–95.00)	91.25 (73.75–94.25)	0.679
Inhaled corticosteroid use, (%)	12	19	0.662
CD4 T-cell count, cell/mm^3^	435.00 (355.00–590.00)	-	-
HIV viral load undetectable, (%)	92	-	-
ART, (%)	96	-	-
Hypertension, (%)	20	44	0.161
Diabetes, (%)	8	19	0.362

Discrete variables are presented as percentages, and corresponding *p*-values were obtained using Fisher exact tests. Continuous variables are presented as median and interquartile range, *p*-values represent Kruskal–Wallis *p*-value. PLHW—people living with human immunodeficiency virus; BMI—body mass index; FVC—forced vital capacity; FEV_1_—forced expiratory volume in 1 s; COPD—chronic obstructive pulmonary disease; BAL—bronchoalveolar lavage; ART—antiretroviral therapy.

**Table 2:** Age- and HIV-associated modules.

Module	#CpGs	#Genes	Effect					
			Age			HIV		
			*p*-Value	FDR	R	*p*-Value	FDR	*p*-Value Age-Adj
Skyblue	412	426	1.89 × 10^−5^	0.001	0.56	0.001	0.002	0.056
Red	2233	1150	1.44 × 10^−4^	0.006	0.55	4.50 × 10^−4^	0.002	0.044
Yellow	2394	1900	2.29 × 10^−4^	0.006	−0.61	0.001	0.002	0.127
Palevioletred3	142	150	2.54 × 10^−3^	0.043	−0.46	0.003	0.006	0.103
Pink	1579	1346	3.38 × 10^−3^	0.043	−0.45	0.007	0.007	0.151
Floralwhite	210	163	2.84 × 10^−3^	0.043	0.45	0.007	0.007	0.173

**Table 3:** Top 5 differentially methylated CpG sites (DMPs) associated with HIV in BAL cells.

Probe	Chr	*p*	*FDR*	Beta Difference (Reference Group: HIV-Uninfected)	Relation to CpG Island	Gene Symbol
Criteria: Lowest FDR						
cg26126053	10	1.43 × 10^−22^	1.12 × 10^−16^	−0.011	Open Sea	DHX32
cg00401660	3	8.05 × 10^−18^	3.17 × 10^−12^	0.018	Open Sea	CPN2
cg14118535	1	7.17 × 10^−17^	1.88 × 10^−11^	−0.013	Open Sea	FUBP1
cg08589141	19	2.00 × 10^−16^	3.94 × 10^−11^	0.029	North Shore	RGL3
cg16636316	11	3.52 × 10^−16^	5.55 × 10^−11^	0.034	Open Sea	OTOG
Criteria: Greatest Beta Difference						
cg09733528	2	3.40 × 10^−7^	9.04 × 10^−4^	−0.316	Open Sea	ABCB11
cg02630646	2	6.08 × 10^−5^	2.35 × 10^−2^	−0.314	Open Sea	--
cg11424828	8	4.02 × 10^−4^	6.82 × 10^−2^	0.273	Island	MYOM2
cg21015022	7	4.07 × 10^−5^	1.87 × 10^−2^	0.254	South Shore	PARP12
cg17975832	12	7.01 × 10^−4^	9.21 × 10^−2^	−0.250	Open Sea	7SK

FDR—false discovery rate. Chr—chromosome.

## Data Availability

DNA methylation data are deposited at the Gene Expression Omnibus repository (GSE262656).

## Supplementary Materials

The following supporting information can be downloaded at: https://www.mdpi.com/article/10.3390/biomedicines12061261/s1, Figure S1: Covariate selection; Figure S2: Volcano plot comparing Alu methylation in individuals with COPD to the reference group, individuals without COPD. There was no pattern of global Alu hypomethylation found in COPD. Blue = hypomethylation in individuals with COPD compared to individuals without COPD; Red = hypermethylation in individuals with COPD compared to individuals without COPD. Statistical significance was set at FDR < 0.10; Figure S3: Volcano plot comparing LINE-1 methylation in individuals with COPD to the reference group, individuals without COPD. There was no pattern of global LINE-1 hypomethylation found in COPD. Blue = hypomethylation in individuals with COPD compared to individuals without COPD; Red = hypermethylation in individuals with COPD compared to individuals without COPD. Statistical significance was set at FDR < 0.10; Table S1: Co-methylation networks; Table S2: Co-methylation networks associated with age; Table S3: Differentially methylated CpGs associated with HIV; Table S4: Differentially methylated Kyoto Encyclopedia of Genes and Genomes (KEGG) pathways; Table S5: Differentially methylated genes overlapping between airway epithelial cells and BAL cells in PLWH; Table S6: Differentially methylated pathways that overlap between the airway epithelial cells and BAL cells of PLWH.
