# Supplementary material for: DNA Methylation Demonstrates Bronchoalveolar Cell Senescence in People Living with HIV: An Observational Cohort Study
Source: Biomedicines. 2024 Jun 6;12(6):1261. doi: 10.3390/biomedicines12061261 (PMC11201658; doi:10.3390/biomedicines12061261)
Supplement: Supplementary file 1 [file biomedicines-12-01261-s001.zip › SupplementaryFigures.pdf]

DNA Methylation Demonstrates Bronchoalveolar Cell Senescence in People Living with HIV: An Observational Cohort Study

Supplementary Figures

Ana I. Hernandez Cordero [1,2], Xuan Li [1], Julia Yang [1], Chen Xi Yang [1], Tawimas Shaipanich [3], Julie L. MacIsaac [4], Kristy Dever [4], Michael S. Kobor [2,4], Julio Montaner [5,6], Marianne Harris [5,7], Silvia Guillemi [5,7], Shu Fan Paul Man [1], Don D. Sin [1,2,3] and Janice M. Leung [1,2,3,*]

[1] Centre for Heart Lung Innovation, St. Paul's Hospital, University of British Columbia, Vancouver, BC V6Z 1Y6, Canada
[2] Edwin S. H. Leong Centre for Healthy Aging, University of British Columbia, Vancouver, BC V6T 1Z3, Canada
[3] Division of Respiratory Medicine, Department of Medicine, Faculty of Medicine, University of British Columbia, Vancouver, BC V6Z 1Y6, Canada
[4] Centre for Molecular Medicine and Therapeutics, University of British Columbia, Vancouver, BC V6H 0B3, Canada
[5] British Columbia Centre for Excellence in HIV/AIDS, Vancouver, BC V6Z 1Y6, Canada
[6] Department of Medicine, Faculty of Medicine, University of British Columbia, Vancouver, BC V6Z 1Y6, Canada
[7] Department of Family Practice, Faculty of Medicine, University of British Columbia, Vancouver, BC V6Z 1Y6, Canada
* Correspondence: janice.leung@hli.ubc.ca

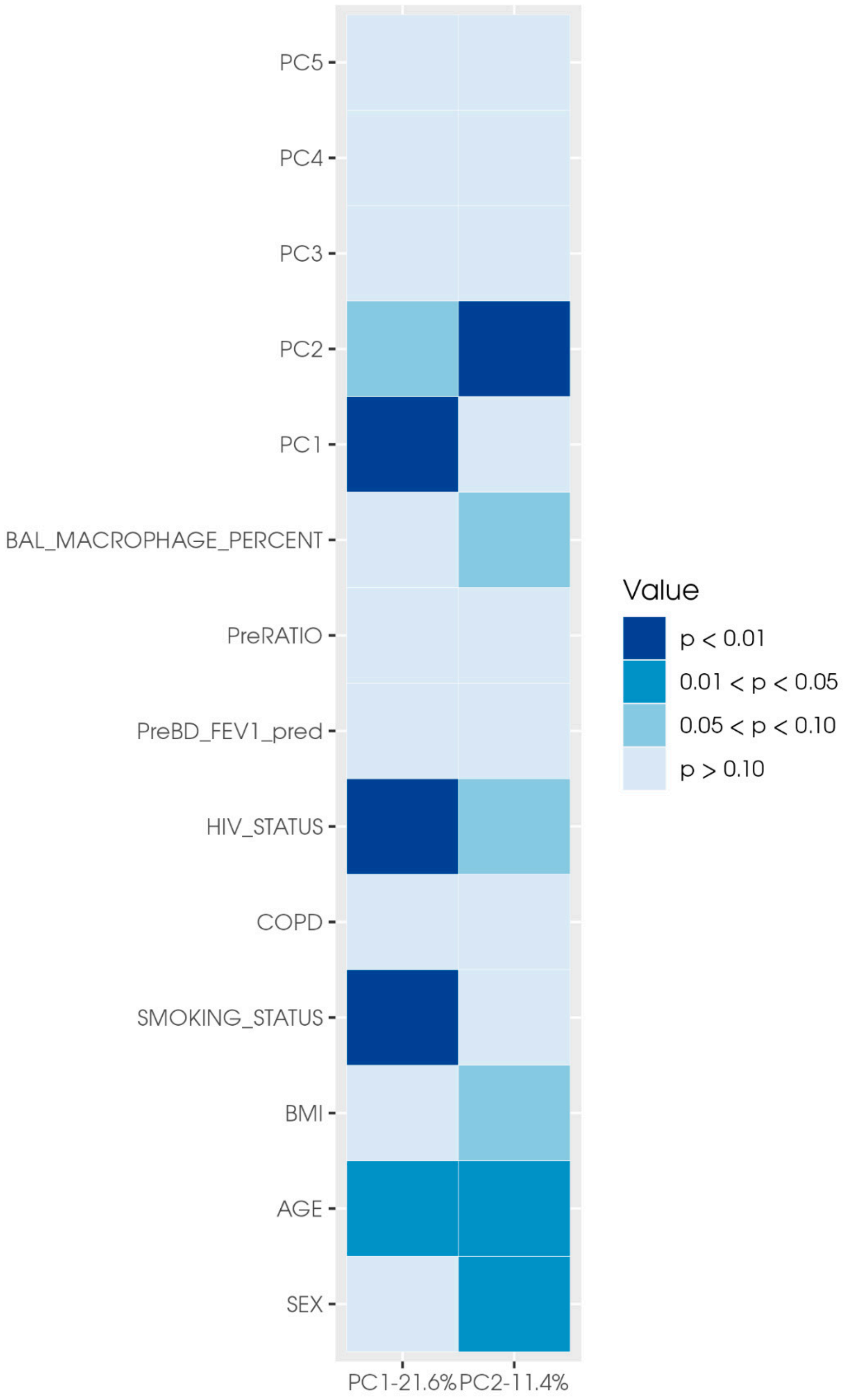

Figure S1. Covariate selection

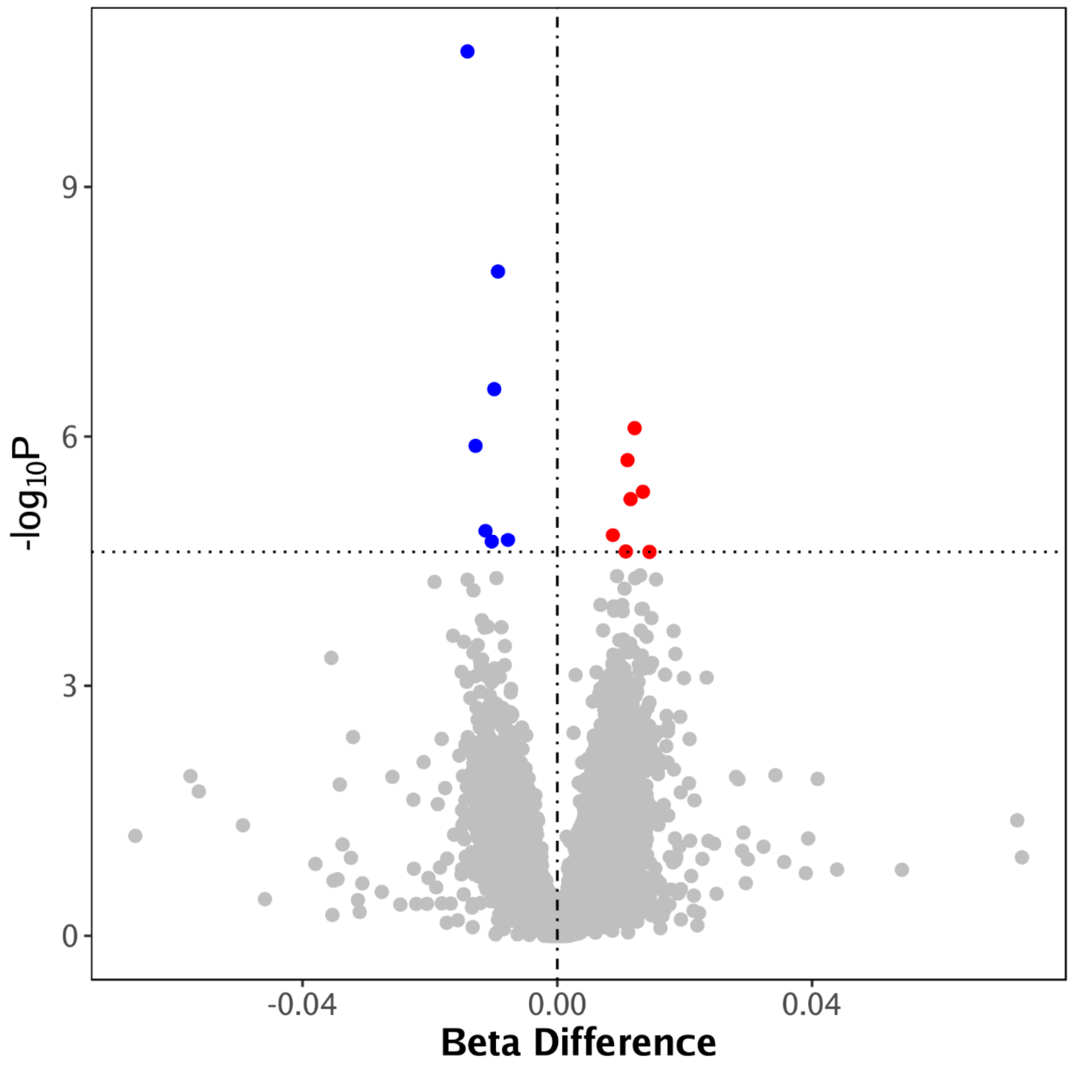

Figure S2. Volcano plot comparing Alu methylation in individuals with COPD to the reference group, individuals without COPD. There was no pattern of global Alu hypomethylation found in COPD. Blue = hypomethylation in individuals with COPD compared to individuals without COPD; Red = hypermethylation in individuals with COPD compared to individuals without COPD. Statistical significance was set at FDR<0.10.

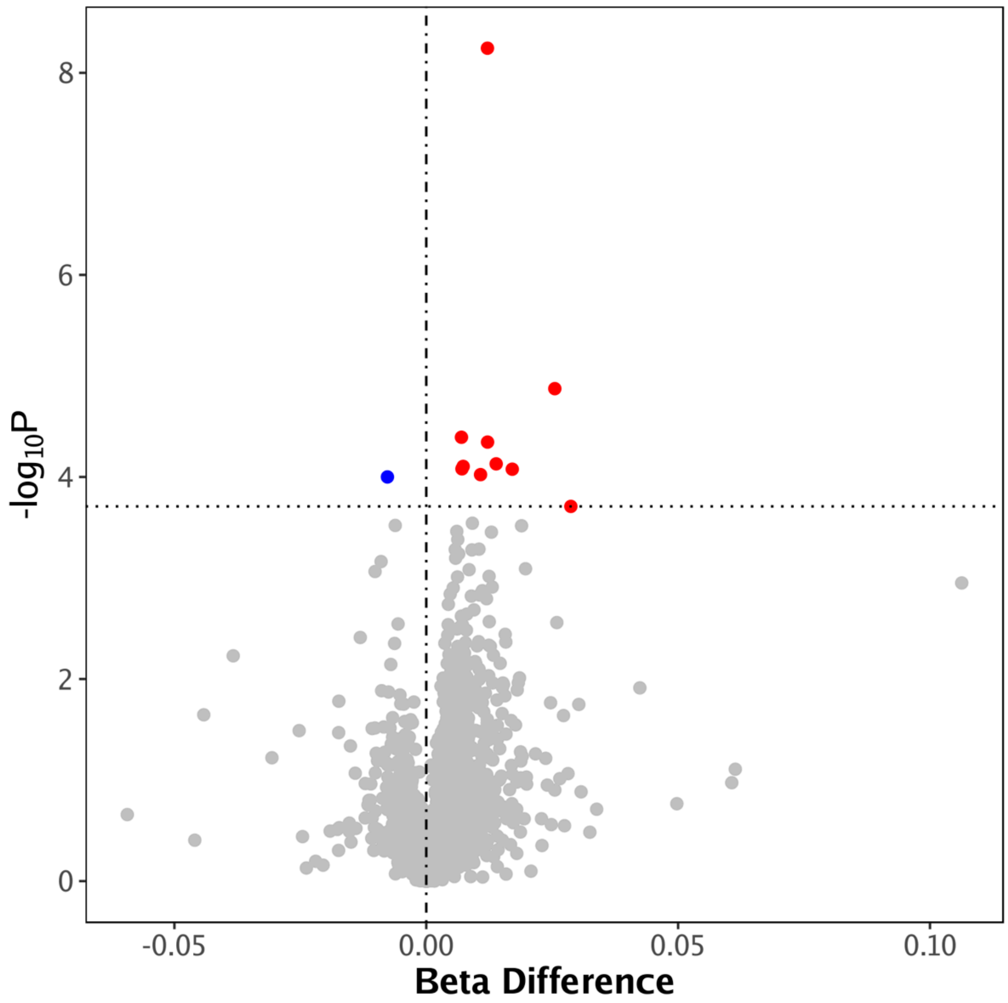

Figure S3. Volcano plot comparing LINE-1 methylation in individuals with COPD to the reference group, individuals without COPD. There was no pattern of global LINE-1 hypomethylation found in COPD. Blue = hypomethylation in individuals with COPD compared to individuals without COPD; Red = hypermethylation in individuals with COPD compared to individuals without COPD. Statistical significance was set at FDR<0.10.
